# Early extubation after elective surgical aortic valve replacement during the COVID-19 pandemic

**DOI:** 10.1186/s13019-024-02989-0

**Published:** 2024-08-24

**Authors:** Anna Fischbach, Julia Alexandra Simons, Steffen B. Wiegand, Lieselotte Ammon, Rüdger Kopp, Gernot Marx, Rolf Rossaint, Payam Akhyari, Gereon Schälte

**Affiliations:** 1 Department of Anesthesiology, RWTH Aachen University, 52074 Aachen, Germany; 2 Department of Anesthesiology and Intensive Care Medicine, Hannover Medical school, 30625 Hannover, Germany; 3 Department of Intensive Care Medicine, RWTH Aachen University, 52074 Aachen, Germany; 4 Department of Cardiothoracic Surgery, RWTH Aachen University, 52074 Aachen, Germany

**Keywords:** Aortic valve surgery, Surgical aortic valve replacement, Early extubation, Enhanced recovery after cardiac surgery, ERACS

## Abstract

**Background:**

Surgical aortic valve replacement (SAVR) is an established therapy for severe calcific aortic stenosis. Enhanced recovery after cardiac surgery (ERACS) protocols have been shown to improve outcomes for elective cardiac procedures. The COVID-19 pandemic prompted early extubation post-elective surgeries to preserve critical care resources.

**Aim of this study:**

To investigate the effects of extubating patients within 6 h post-elective SAVR on hospital and ICU length of stay, mortality rates, ICU readmissions, and postoperative pneumonia.

**Study Design and methods:**

The retrospective analysis at the University Hospital Aachen, Germany, includes data from 2017 to 2022 and compares a total of 73 elective SAVR patients. Among these, 23 patients were extubated within 6 h (EXT group), while 50 patients remained intubated for over 6 h (INT group).

**Results:**

The INT group experienced longer postoperative ventilation, needed more vasopressor support, had a higher incidence of postoperative pneumonia, and longer ICU length of stay. No significant differences were noted in overall hospital length of stay, mortality, or ICU readmission rates between the groups.

**Conclusion:**

This study demonstrates that early extubation in high-risk, multimorbid surgical aortic valve replacement patients is safe, and is associated with a reduction of pneumonia rates, and with shorter ICU and hospital length of stays, reinforcing the benefits of ERACS protocols, especially critical during the COVID-19 pandemic to optimize intensive care use.

**Supplementary Information:**

The online version contains supplementary material available at 10.1186/s13019-024-02989-0.

## Introduction

Surgical aortic valve replacement (SAVR) and transcatheter aortic valve implantation (TAVI) have emerged as the mainstays of treatments for severe calcific aortic stenosis (AS), offering improvement of symptoms and a survival advantage to affected patients [1, 2]. The collaborative efforts of a multidisciplinary heart valve team, including cardiologists, structural valve interventionalists, cardiovascular surgeons, anaesthesiologists, and nurses, are crucial in planning treatment that addresses the unique needs and preferences of patients with severe AS requiring valve replacement [3–5]. This comprehensive approach ensures consideration of factors such as the patient’s life expectancy, comorbidities, anatomical characteristics, and personal values. Current guidelines strongly advocate for aortic valve replacement in patients with severe AS who present with cardiac-related symptoms such as dyspnoea, syncope, or angina pectoris. In contrast, the recommendation is weaker for asymptomatic individuals [5]. The urgency of treatment becomes apparent when considering that untreated symptomatic patients face a 4-year mortality rate close to 50% [6]. Hence, for patients with a life expectancy exceeding one year and an anticipated improvement in quality of life, the choice between SAVR and TAVI is important. SAVR is preferred for symptomatic aortic valve stenosis, while TAVI is recommended for those at elevated surgical risk [4, 5].

The concept of Enhanced recovery after surgery (ERAS), and its cardiac-specific equivalent, enhanced recovery after cardiac surgery (ERACS), embodies a multi-modal, multidisciplinary strategy aimed at optimizing perioperative care to facilitate faster recovery post-surgery [7–12]. These protocols, designed to mitigate surgical and anaesthetic stress responses, employ diverse analgesic techniques for postoperative pain management, to promote early extubation, reduce hospital stay durations, expedite patient recovery, decrease the incidence of postoperative complications, and, ultimately, improve clinical outcomes. Evidence supports that ERACS protocols contribute to earlier postoperative extubation, diminished reliance on opioids post-surgery, and reduced lengths of stay in both the ICU and the hospital. Furthermore, studies have documented reduced postoperative complication rates, enhanced pain control, and lower overall healthcare costs [12–16]. The COVID-19 pandemic placed unprecedented demands on healthcare resources, particularly ICU capacities, exacerbating the challenges faced by healthcare facilities. In response, healthcare providers implemented strategies to alleviate system burdens, especially regarding elective surgeries. Protocols emphasizing rapid post-surgery extubation were adopted to preserve critical ICU resources for COVID-19 patients [17, 18].

Considering these developments, our retrospective study focused on patients who underwent elective SAVR during the COVID-19 pandemic and were extubated within 6 h post-surgery. The study aimed to assess the impact of early extubation on various outcomes, including the LOS in the ICU and hospital, ICU readmission rates, the incidence of postoperative pneumonia, and mortality rates in both the ICU and hospital settings. The aim of this study is to provide insights into the benefits of early extubation practices in a multi-morbid patient population and its safety and feasibility.

## Materials and methods

We reviewed clinical records from 2017 to 2022 of patients who underwent elective SAVR at the department of cardiac surgery, at the University Hospital Aachen, Germany. Ethical approval for the study was obtained from the local ethics committee (protocol number EK 22–410, EK 22-371, EK 24-101). The approvals were granted on December 1st, 2022, December 18th, 2023, and March 07th, 2024 respectively, thereby authorizing the inclusion of patient data from two distinct periods: 2017–2021 and from 2022.

### Patients

Patients meeting the following inclusion criteria were considered eligible: (1) aged ≥ 18 years and (2) undergoing elective SAVR for the treatment of aortic valve stenosis. Elective surgery was classified as non-emergency aortic valve replacement surgery. Patients were excluded from the study, if they met any of the following exclusion criteria: (1) NSTEMI or STEMI shortly prior to aortic valve replacement; (2) presence of other cardiac pathologies such as aortic aneurysm or mitral valve insufficiency, (3) aortic valve replacement combined with other surgical procedures such as coronary artery bypass (CABG) surgery.

Between 2017 and 2022, a total of 256 patients undergoing aortic valve replacement for acquired aortic stenosis at University Hospital Aachen were initially considered for this study. Of these, exclusions were made as follows: 15 patients were excluded due to incomplete medical records, specifically missing ICU records or anaesthesia protocols. An additional 32 patients were excluded for undergoing minimally invasive aortic valve replacement surgery, and 70 patients were excluded because they received concurrent cardiac procedures in addition to SAVR. Moreover, 3 patients were excluded due to the presence of other significant cardiac pathologies, and 2 patients were excluded for requiring immediate postoperative ECMO support following SAVR. After these exclusions, 134 patients remained eligible for the study. This cohort was further categorized into two groups based on the duration of postoperative intubation: 89 patients remained intubated for 6 or more hours following SAVR (INT group), while 45 patients were extubated either immediately or within 6 h post-SAVR (EXT group). Within the INT group, 39 patients were subsequently excluded due to undergoing SAVR during the COVID-19 pandemic. In the EXT group, 21 patients were excluded for receiving SAVR prior to the pandemic, and an additional patient was excluded for having congenital aortic valve stenosis. Finally, 50 patients were included in the INT group and 23 patients in the EXT group.

### Study groups

The study population was divided into two groups based on the timing of extubation following SAVR. The first group, designated as the EXT group, comprised patients who were extubated within the initial 6 h post-surgery (< 6 h). In contrast, the second group (INT group), included patients whose extubation occurred beyond 6 h post-surgery (≥ 6 h). The anesthesia regimen in cardiothoracic surgery is standardized. For induction, patients received sufentanil, propofol, and rocuronium, while sevoflurane and sufentanil were used for maintenance. The INT cohort exclusively consisted of patients treated between 2017 and 2019, a period preceding the COVID-19 pandemic, while the EXT group included patients during the COVID-19 pandemic (2020 to 2022).

Criteria for early extubation during the pandemic were defined as follows: patients were considered eligible if they met the general extubation criteria [19], demonstrated hemodynamic stability (norepinephrine doses < 0.2 µg/kg/min and/or epinephrine doses < 0.1 µg/kg/min), if they did not had more than 200 ml of blood drainage from the chest tube, and if they had a successful “wake-up” trial during which the neurological function, the compliance, and respiratory recovery was assessed.

**Fig. 1:**
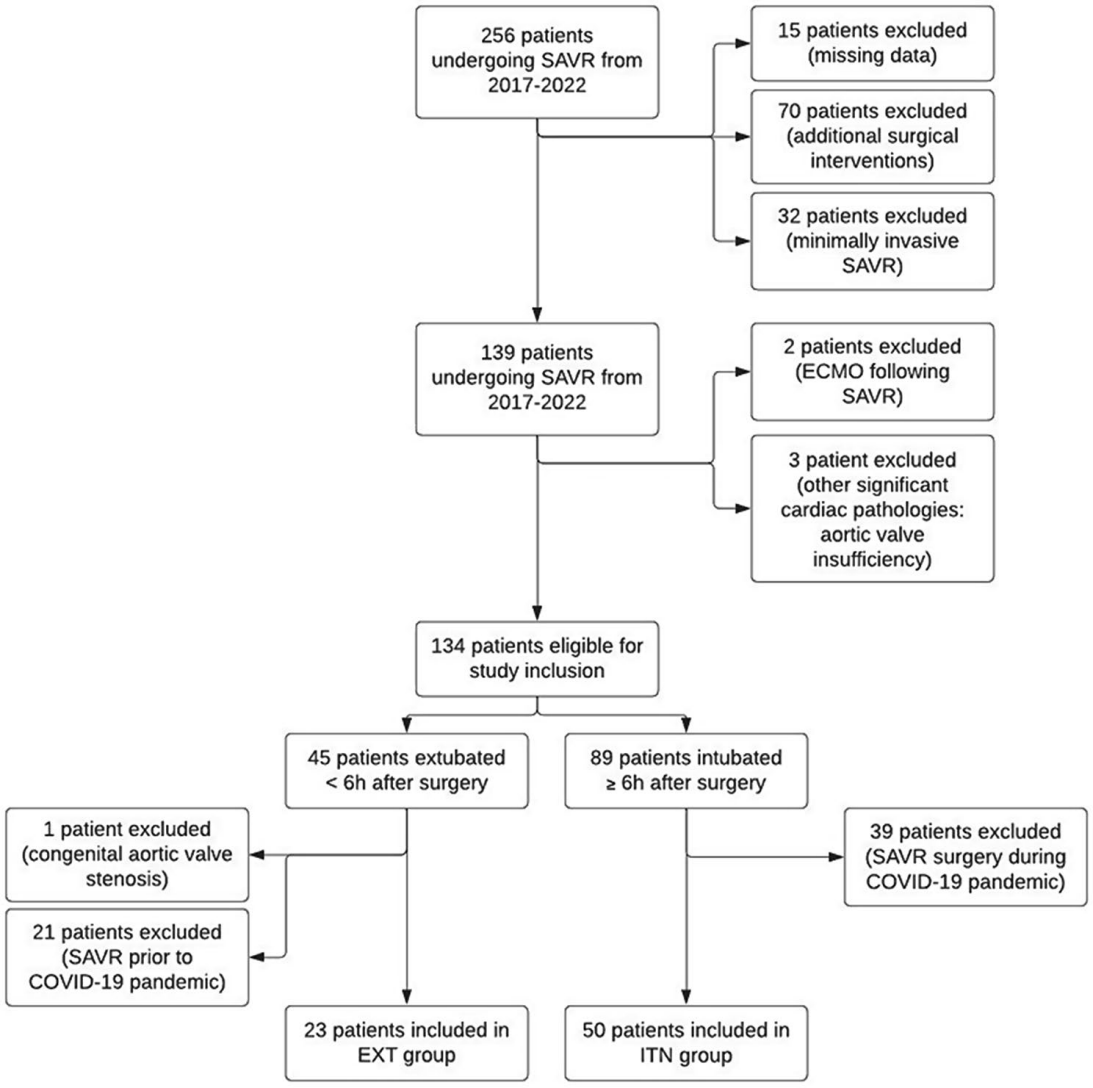
Selection of patients undergoing SAVR from 2017 to 2022

To facilitate a rigorous and statistically valid comparison between the two groups, patients in the INT cohort were matched with those in the EXT cohort based on key baseline clinical characteristics, including age, body mass index (BMI), and sex, as well as prevalent comorbidities such as presence of coronary artery disease, diabetes mellitus or a pulmonary disease (co-variate matching). The Sequential Organ Failure Assessment (SOFA) and Euro SCORE II were also included in the matching process to further enhance a comparability of the two groups. The patient selection process is shown in Fig. 1.

### Study end points

The co-primary outcomes were (1) ICU length of stay (LOS); (2) ICU readmission and (3) ICU mortality. Secondary outcomes were (1) hospital LOS, (2) hospital mortality and (3) incidence of postoperative pneumonia.

### Statistical analysis

Statistical analysis and graph designs were performed using GraphPad Prism software (Version 9.3.1, GraphPad Software, San Diego, CA, USA). Significance was determined at a p-value threshold below 0.05. Data were represented as median (first quartile – third quartile) or percentages. Assessment of normal distribution was conducted using the Shapiro-Wilk test. If the data were normally distributed, an unpaired t-test was applied. If the data were not normally distributed, a Mann-Whitney test was used. The association of parameters with primary or secondary outcomes were also calculated in time-depending Cox regression model, in which case hazard ratios (HR) (Mantel-Haenszel) were calculated. Kaplan-Meier analysis was used to visualize both groups.

The Absolute Standardized Difference (ASD) was calculated to quantify the effect size and compare the baseline characteristics between the EXT and INT groups. The ASD was determined using the following formula:

$$\:ASD=\:\frac{|{\stackrel{-}{X}}_{EXT}-{\stackrel{-}{X}}_{INT}|}{{SD}_{pooled}}$$

Where $\:{\stackrel{-}{X}}_{EXT}-{\stackrel{-}{X}}_{INT}$ are the means of the EXT and INT groups, respectively, and SD_pooled_ is the pooled standard deviation. The pooled standard deviation was calculated as:

$$\:{SD}_{pooled\:}=\:\sqrt{\frac{({{SD}^{2}}_{EXT}+\:{{SD}^{2}}_{INT})}{2}}$$

For every statistical analysis conducted, we included all patients. If it was not possible to include every patient due to missing data, we indicated it in the description below the table or figure. A comprehensive literature review was conducted using multiple databases, including PubMed, MEDLINE, Cochrane Library, Embase, and Scopus.

## Results

### Baseline, surgical and postoperative clinical characteristics of patients undergoing elective SAVR

73 patients fulfilled the inclusion criteria and were included in this study. The majority of patients were male (62%) and had a median age of 66 years (63–72 years) as shown in Table 1. When comparing the clinical characteristics of patients who were extubated within 6 h after surgery (EXT group) with those who remained intubated for more than 6 h after surgery (INT group), both groups did not show significant differences, except for the length of surgery, the duration of invasive mandatory ventilation (i-MV) and the duration of vasopressor support after the surgery. The length of surgery was significantly longer in the EXT group compared to the INT group (4.5 h (4–5 h) vs. 5 h (5–6 h); *p* < 0.01). The postoperative i-MV duration was significantly longer in the INT group, in contrast to the EXT group (11 h (9–16 h) vs. 0.5 h (0–4 h); *p* < 0.01). The postoperative vasopressor support was significantly longer in the INT group as opposed to the EXT group (2 h (2–4 h) vs. 2 h (1–2 h); *p* < 0.01).

**Table 1:** Baseline clinical characteristics of patients undergoing SAVR surgery

Characteristic	All Patients *n* = 73	Intubated ≥ 6 h after surgery (INT) *n* = 50	Extubated < 6 h after surgery (EXT) *N* = 23	ASD	*p* Value
**Baseline characteristics**					
Age, y	66 (63–72)	67 (63–74)	65 (62–69)	0.408	0.14
Male sex	45 (62%)	31 (62%)	14 (61%)		> 0.99
BMI, kg/m^2^	28 (25–32)	27 (24–31)	29 (27–32)	0.210	0.21
SVCAD	13 (18%)	9 (18%)	4 (17%)		0.96
DVCAD	4 (5%)	3 (6%)	1 (4%)		> 0.99
TVCAD	2 (3%)	2 (4%)	0 (0%)		0.56
Diabetes mellitus	15 (21%)	10 (20%)	5 (22%)		> 0.99
Pulmonary disease	12 (16%)	10 (20%)	2 (9%)		0.32
Pathological pulmonary function test ^1^	15 (21%)	10 (20%)	5 (22%)		> 0.99
Left ventricular ejection fraction (%)	60 (53–60)	60 (54–60)	60 (53–60)	0.215	0.46
EURO II score ^2^, %	1.12 (0.88–1.62)	1.14 (0.89–1.62)	1.02 (0.81–1.69)	0.242	0.74
SOFA Score	1 (0–1)	1 (0–1)	0 (0–1)	0.259	0.21
**Surgical characteristics**					
Mechanical aortic valve prosthesis	12 (16%)	9 (18%)	3 (13%)		0.74
Biological aortic valve prosthesis	61 (84%)	41 (82%)	20 (87%)		0.74
Minimally invasive aortic valve surgery	19 (26%)	10 (20%)	9 (39.1%)		0.10
Open aortic valve surgery	54 (74%)	40 (80%)	14 (60.9%)		0.10
Length of surgery, h	5 (4-5.5)	4.5 (4–5)	5 (5–6)	0.780	< 0.01*
CBP duration, min	97 (82–118)	90 (76–106)	113 (93–143)	0.938	< 0.01*
Aortic cross-clamp duration, min ^3^	69 (58–87)	66 (55–73)	86 (69–106)	1.275	< 0.01*
Perioperative complications	36 (49%)	25 (50%)	11 (48%)		> 0.99
**Postoperative characteristics**					
Duration of vasopressor support, d	2 (2–3)	2 (2-3.75)	2 (1–2)	0.493	< 0.01*
Duration i-MV, h ^4^	9 (4–13)	11.25 (8.63–15.88)	0.5 (0–4)	0.436	< 0.01*

When comparing the clinical characteristics of patients who were extubated within 6 h after surgery (EXT group) with those who remained intubated for more than 6 h after surgery (INT group), both groups did not show significant differences, except for the length of surgery, the duration of invasive mandatory ventilation (i-MV) and the duration of vasopressor support after the surgery

Results are shown as median (first quartile-third quartile) or No. (%)

ASD = absolute standardized difference; BMI = body mass index; SVCAD/DVCAD/TVCAD = single/double/three vessel coronary artery disease; CPB = cardio-pulmonary-bypass; Euro II SCORE = European System for Cardiac Operative Risk Evaluation; SOFA score = Sequential Organ Failure Assessment score; i-MV = invasive mandatory ventilation

^1^ pathological if severe obstructive or restrictive pulmonary disease was detected

^2^ for the INT group, only 49 out of 50 were included in the analysis for the EURO II score due to missing data

^3^ cross-clamp time could not be determined for 5 out of 23 in the EXT group and for 10 out of 50 in the INT group due to missing data

^4^ the ventilation time during the operation is not included in the stated ventilation time

### Postoperative complications and clinical characteristics 24 h after elective SAVR

**Fig. 2:**
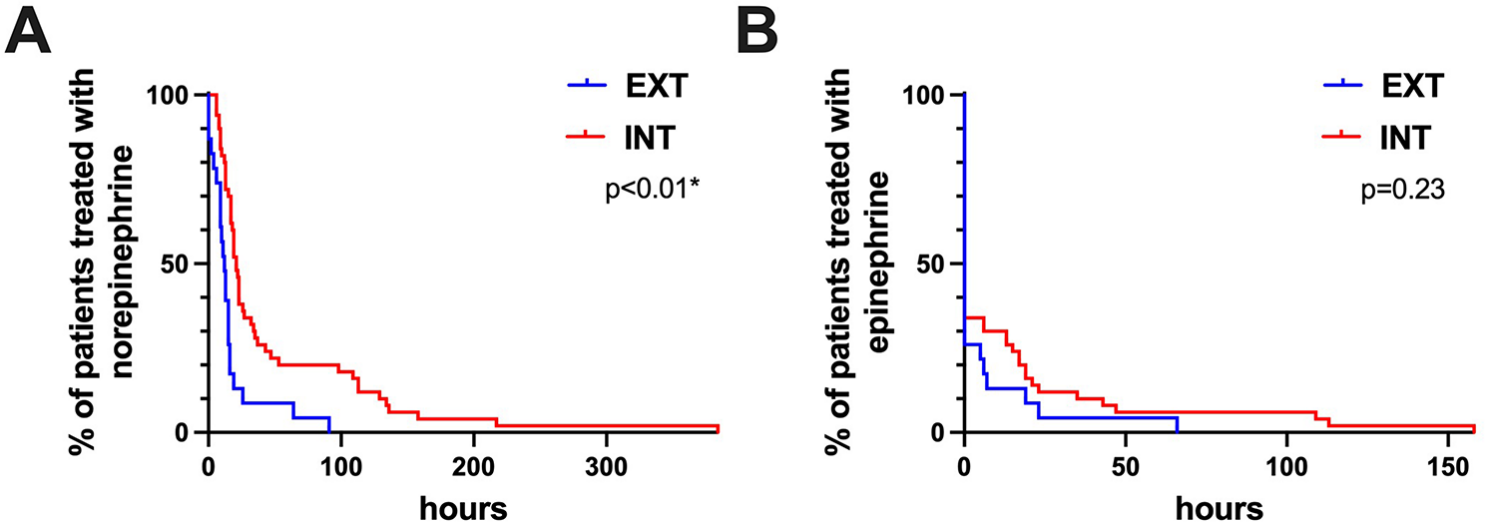
Percentage (%) of patients in the EXT and INT group with duration of norepinephrine administration (**A**) and duration of epinephrine administration (**B**). The INT group had a 3.6 higher risk for a continued norepinephrine administration compared to the EXT group (*p* < 0.01; HR: 3.6; CI: 1.9–7.0). There was no significant difference between both groups in the duration of administration of epinephrine (*p* = 0.23; HR: 1.6; CI: 0.7–3.5)

There was no significant difference between both groups in clinical characteristics at 24 h after the surgery nor in the occurrence of postoperative complications or rate of re-intubations (Table 2). Patients in the INT group did not require a higher dosage of norepinephrine 24 h post-surgery compared to patients of the EXT group (*p* = 0.05). However, the Log-rank test revealed that the INT group had a 3.6 higher risk for a continued norepinephrine administration compared to the EXT group (*p* < 0.01; HR: 3.6; CI: 1.9–7.0) (Fig. 2(A)). When comparing the means of epinephrine administration between the two groups, no significant difference was observed (*p* = 0.10). Similarly, the log-rank analysis did not show a significant difference between the groups (*p* = 0.23; HR: 1.6; CI: 0.7–3.5) (Fig. 2(B)).

**Table 2:** Postoperative complications and clinical characteristics 24 h after SAVR

	All Patients *n* = 73	Intubated ≥ 6 h after surgery (INT) *n* = 50	Extubated < 6 h after surgery (EXT) *n* = 23	*P* Value
Characteristic				
24 h after surgery				
PaO_2_/FiO_2_, mmHg	275 (224–308)	275 (227–311)	264 (222–301)	0.83
paCO_2_, mmHg	40.7 (38-42.6)	40.5 (37.8–43)	41 (38.6–42.2)	0.85
MAP, mmHg	73 (66–84)	72 (65–83)	75 (70–87)	0.07
HR, beats per minute	86 (78–90)	89 (80–90)	82 (75–90)	0.12
Use of norepinephrine, ug/kg/min	0 (0-0.005)	0 (0-0.013)	0 (0–0)	0.05
Use of epinephrine, ug/kg/min	0 (0–0)	0 (0–0)	0 (0–0)	0.10
Postoperative complications				
Bleeding^1^	6 (8%)	6 (12%)	0 (0%)	0.17
Pleural effusion	28 (38%)	22 (44%)	6 (26%)	0.20
Delirium	15 (21%)	13 (26%)	2 (9%)	0.12
Atrial fibrillation	32 (44%)	23 (46%)	9 (39%)	0.62
Other^2^	24 (33%)	20 (40%)	4 (17%)	0.07
Reintubation^3^	5 (7%)	5 (10%)	0 (0%)	0.17

There was no significant difference between both groups in clinical characteristics at 24 h after the surgery nor in the occurrence of postoperative complications or rate of re-intubations

Results are shown as median (first quartile-third quartile) or No. (%)

MAP = mean arterial pressure; HR = heart rate

^1^ 2 out of 6 patients received a mechanical aortic valve prosthesis

^2^ other postoperative complication include pneumothorax, brady - or tachyarrhythmias other than atrial fibrillation, cardiogenic or septic shock

^3^ reintubation due to partial or global respiratory insufficiency. None of these 5 patients needed re-intubation within the first 24 h after extubation

### Primary and secondary outcomes

**Fig. 3:**
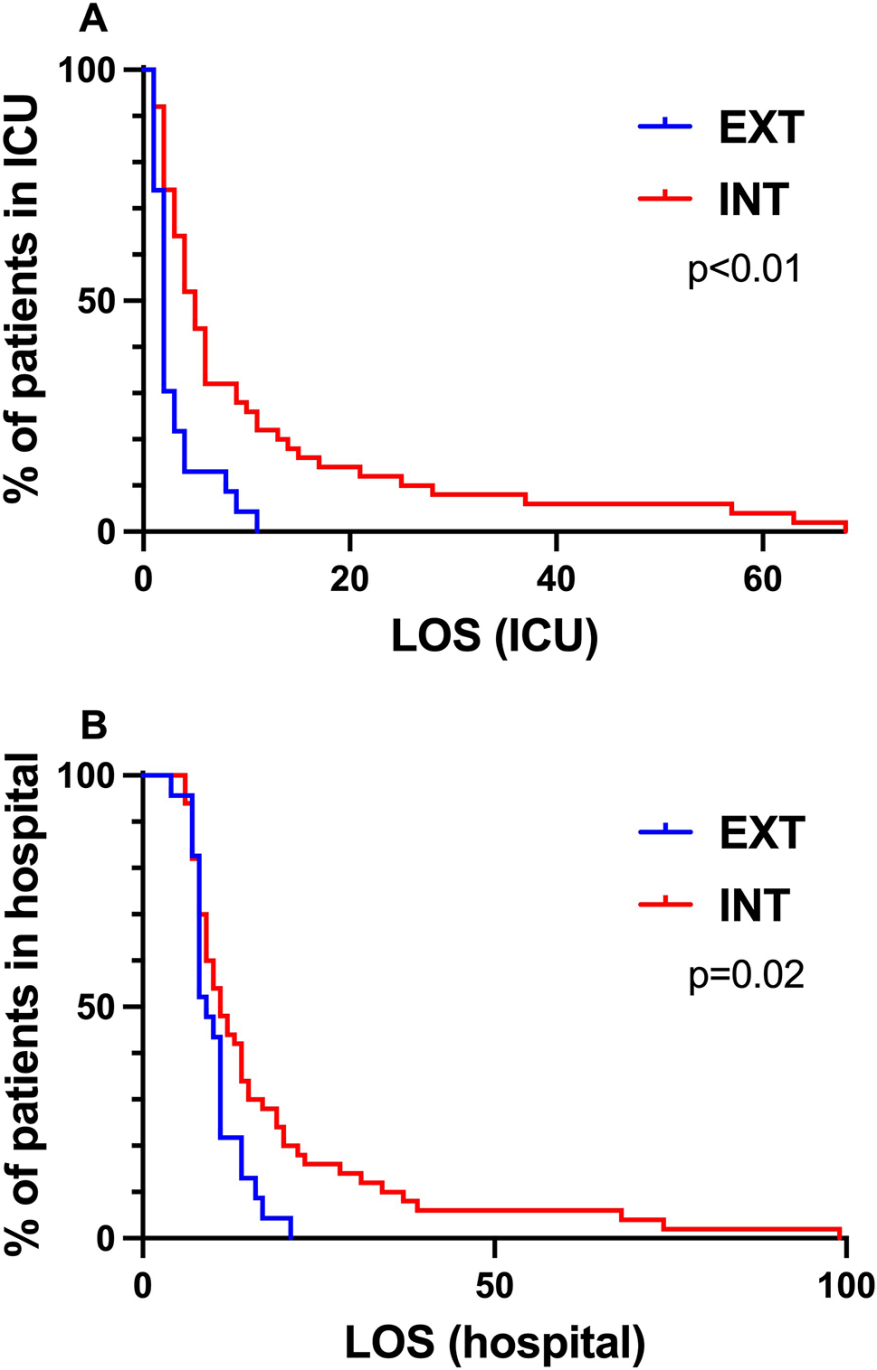
ICU and hospital LOS of patients in the EXT and INT group. (**A**) ICU LOS of patients in the EXT and INT group. (**B**) Hospital LOS of patients in the EXT and INT group. Patients intubated for 6 h or longer following surgery had a 4.1-fold higher risk of prolonged ICU stays (*p* < 0.01; HR: 4.1; CI: 2.0–8.3) and a 2.1 times higher increased risk for a prolonged stay in the hospital (*p* = 0.02; HR: 2.1; CI: 1.1–3.9). LOS = length of stay; ICU = intensive care unit

Patients who remained intubated for more than 6 h after the surgery experienced a significantly higher occurrence of postoperative pneumonia (18% vs. 0%; *p* < 0.05) as opposed to patients who were extubated within 6 h after surgery (Table 3). Patients within the INT group had a significantly extended ICU LOS compared to those in the EXT group (5d (2-11d) vs. 2d (2-3d); *p* < 0.01). Patients intubated for 6 h or longer following surgery had a 4.1-fold higher risk of prolonged ICU stays (*p* < 0.01; HR: 4.1; CI: 2.0–8.3) (Fig. 3(A)) and a 2.1 times increased risk for a prolonged stay in the hospital (*p* = 0.02; HR: 2.1; CI: 1.1–3.9) (Fig. 3 (B)). Patients diagnosed with pneumonia experienced a significantly longer LOS in both the ICU (*p* < 0.01) and the hospital (*p* < 0.01) (Supplement Table 1). There was no statistically significant difference in ICU readmissions rates between both groups.

**Table 3:** Primary and secondary outcomes

	All Patients *n* = 73	Intubated ≥ 6 h after surgery (INT) *n* = 50	Extubated < 6 h after surgery (EXT) *n* = 23	*P* Value
Primary outcomes				
ICU LOS, median (first quartile-third quartile)	4 (2–9)	5 (2–11)	2 (1.5-3)	< 0.01*
ICU readmissions, n (%)	17 (23%)	15 (30%)	2 (9%)	0.07
ICU mortality, n (%)	1 (1%)	1 (2%)	0 (0%)	> 0.99
Secondary outcomes				
Hospital LOS, median (first quartile-third quartile)	11 (8–15)	11 (8–19)	9 (8–11)	0.10
Hospital mortality, n (%)	1 (1%)	1 (2%)	0 (0%)	> 0.99
Postoperative pneumonia, n (%) ^1^	9 (12%)	9 (18%)	0 (0%)	< 0.05*

Patients within the INT group had a significantly extended ICU LOS and experienced a significantly higher occurrence of postoperative pneumonia compared to those in the EXT group

Results are shown as median (first quartile-third quartile) or No. (%)

ICU = intensive care unit; LOS = length of stay

^1^ 6 out of 9 patients (67%) developed pneumonia in the first postoperative week. 3 out of 9 patients (33%) developed pneumonia two weeks postoperatively

## Discussion

This study found that patients in the INT group, who were intubated for more than 6 h after surgery, required prolonged postoperative ventilation and vasopressor support, and experienced a higher rate of postoperative pneumonia compared to patients of the EXT group. There was no significant difference in reintubation rates due to respiratory insufficiency. When comparing both groups, the INT group experienced significantly longer ICU length of stay than the EXT group. In addition, patients who were intubated for more than 6 h after surgery were associated with longer hospital stays compared to those who were extubated within 6 h following SAVR. There were no significant differences in mortality rates, or ICU readmission rates between both groups. 

Prolonged intubation after cardiac surgery has been shown to be associated with an increased risk of ventilator-associated pneumonia [20–23], as seen in the INT group of our study. The infection-triggered release of inflammatory factors, causing vasodilation, may explain the extended vasopressor support observed. At the same time, intubation also requires pharmacological sedation, potentially leading to decreased blood pressure and hence, a prolonged need for vasopressors, such as norepinephrine. Patients who develop pneumonia after a complicated surgery are more likely to stay longer in the ICU and in the hospital, an association that could also be demonstrated in the present study. While longer intubation times in post-cardiac surgery have been linked to increased mortality, morbidity, and postoperative delirium in other studies [24, 25], our study did not find a significant increase in delirium or mortality rates. One potential explanation could be the limited number of patients enrolled in our study. It is also important to consider that patients may remain intubated longer in the ICU simply due to a lack of sufficient personnel resources [26]. Furthermore, it is crucial that the extubation criteria are met for this particular patient group before extubation, as a too early extubation and its consequences can lead to increased morbidity and mortality [27].

For the EXT group, we specifically selected patients from the years of the COVID-19 pandemic (2020–2022). This decision was made as there was a notable increase in the incidence of COVID-19 patients requiring ICU admission [17, 18]. In response, our hospital implemented strategies that aimed at reducing ICU stay durations for elective surgeries of multimorbid patients, such as SAVR. One strategy involved the expedited extubation of patients following elective surgery, thereby significantly reducing their postoperative ICU stay compared to the period preceding the COVID-19 pandemic. It is important to clarify that we did not select specifically patients with favourable outcomes within the time frame of the COVID-19 pandemic. Instead, patients were included, who, under normal circumstances, would have remained intubated for a longer duration. For the INT group, patients were selected from the time preceding the COVID-19 pandemic (2017–2019). The extended time frame was necessary due to the limited number of patients meeting the inclusion criteria of this study. While the comparison of the means of the hospital length of stay between the EXT and INT groups using a t-test did not show a significant difference, analysis with the Log Rank Test revealed a significant difference. This discrepancy might be attributed to the relatively small sample size of this study, which could have been insufficient to detect a difference through the t-test. Increasing the number of patients is likely to yield a significant difference in the t-test results as well.

There was a significant difference in surgery times between the two groups, potentially complicating direct comparisons. Despite the statistical significance in surgery duration, 5 h (5–6 h) for the EXT group versus 4.5 h (4–5 h) for the INT group - the actual difference in time was minor. Interestingly, the EXT group, which had slightly longer surgery times on average, did not experience higher rates of pneumonia and extended ICU length of stay that was observed in the INT group. This indicates that surgery duration alone does not explain the differences found in outcomes. Several studies have already shown the benefits of ERACS protocols for both patients and hospitals [28–32] and several guidelines suggest protocols for this approach [7, 11, 33]. Benefits included within the ERACS protocols were reductions in opioid use, and gastrointestinal complications [9], a significant reduction in surgical and medical complications and better pain management [34]. Another study could show that extubation in the operating room compared to extubation within 6 h after CABG surgery leads to a significant reduction in length of hospital stay, resulting in a significant reduction of hospital costs ($29,602 vs. $31,565) [35]. At our hospital, however, ERACS protocols were inconsistently applied in cardiac anaesthesia and cardiac surgery before and during the COVID-19 pandemic. Therefore, the pandemic-induced ICU bed shortages led to reconsideration of early extubation for elective cardiac surgery patients to reduce ICU time [17], a strategy proven effective in this study.

This study focuses on patients who underwent SAVR, a procedure typically performed on older patients with multiple cardiopulmonary pre-existing diseases. The rate of perioperative complications in these surgeries varies based on the age of the patient, pre-existing health conditions, and the expertise of the hospitals in performing such procedures [34, 36, 37]. Perioperative mortality rates are reported to be between 2.2% and 6.1% [38, 39], classifying this surgery as high-risk. Our findings indicate that early extubation in these patients is safe, resulting in a lower incidence of pneumonia and a significant shorter ICU and hospital LOS. Other studies have shown that pro-longed ICU stays are associated with higher mortality rates [40]. However, in this study, we could not find a significant difference in mortality rates. It is also important to consider that there are factors that favour early extubation. Nguyen et al. [41] demonstrated in their retrospective study, which included a total of 8,872 patients undergoing cardiac surgery, that factors such as male sex; a BMI > 30 kg/m2; undergoing isolated valve surgery; and having a fast-track designation, coronary artery disease, and daytime admission to the ICU were associated with earlier extubation compared to other patients. However, a unique aspect of this study is that due to the COVID-19 pandemic and the very limited capacities in the ICU, physicians were advised to extubate patients early to free up beds for the care of COVID-19 patients. Therefore, the likelihood that a patient was not extubated because they were admitted to the ICU at night i.e. is quite unlikely due to the strains on the ICU at that time. However, it needs to be considered that these factors could have influenced the extubation of patients before the COVID-19 pandemic, which cannot be excluded. At the same time, recent studies aim for extubation in the OR [42]. Physicians must consider whether extubation in the OR is truly necessary. This study demonstrated that extubation within 6 h is associated with a significantly shorter ICU LOS, suggesting that patients may not necessarily need to be extubated in the OR.

Our study has several limitations. First, there is the possibility of a selection bias in this study. Patients in the INT group were exclusively chosen from the years 2017–2019, the time period before the COVID-19 pandemic. In contrast, patients in the EXT group were selected solely during the COVID-19 pandemic period (from 2020 to 2022), as early extubation protocols were initiated in our hospital at the onset of the pandemic to reduce the strain on ICU resources. If patients for the INT group would have also been selected during the time frame of the COVID-19 pandemic, it might be possible that patients would be included who required prolonged intubation. Second, we did not perform a power calculation, primarily because we already included all eligible patients for the EXT group from the pandemic period. As our study was conducted at a single center, it was not possible to further increase the sample size of this study. Third, since this study has a retrospective design, we rely on the accurate documentation in the system, which may introduce information bias.

In late 2023, the university hospital where the study was conducted implemented the ERACS program, led by cardiac anaesthesiologists and involving comprehensive onboarding and structural changes across multiple departments, supported by data from this, other studies and data from the medical controlling department. This initiative included developing new standard operating procedures and educational protocols to optimize patient care.

In summary, this study demonstrates that early extubation in high-risk, multimorbid patients is safe, and is associated with a reduction of pneumonia rates, and with shorter ICU and hospital length of stays. To further explore the benefits of early extubation of multimorbid patients in high-risk surgical procedures, multicentric, prospective studies with a larger patient cohort are needed.

### Electronic supplementary material

Below is the link to the electronic supplementary material.

Supplementary Material 1

## Data Availability

The data that support the findings of this study are not openly available due to reasons of sensitivity and are available from the corresponding author upon reasonable request. Data are located in controlled access data storage at the University Hospital Aachen.

## References

[1] Bagur R, Martin GP, Nombela-Franco L, Doshi SN, George S, Toggweiler S, et al. Association of comorbid burden with clinical outcomes after transcatheter aortic valve implantation. Heart. 2018;104(24):2058. 30030336 10.1136/heartjnl-2018-313356

[2] Bendary A, Ramzy A, Bendary M, Salem M. Transcatheter aortic valve replacement in patients with severe aortic stenosis and active cancer: a systematic review and meta-analysis. Open Heart. 2020;7(1):e001131. 32201582 10.1136/openhrt-2019-001131 PMC7066604

[3] Pellikka PA, Padang R, Scott CG, Murphy SME, Fabunmi R, Thaden JJ. Impact of managing provider type on severe aortic stenosis management and mortality. J Am Heart Assoc. 2022;11(13). 10.1161/JAHA.121.025164 PMC9333396 35766279

[4] Vahanian A, Praz F, Milojevic M, Beyersdorf F. The ten commandments for the 2021 ESC/EACTS guidelines on valvular heart disease. Eur Heart J. 2021;42(41):4207–8. 34529759 10.1093/eurheartj/ehab626

[5] Members WC, Otto CM, Nishimura RA, Bonow RO, Carabello BA, Erwin JP, et al. 2020 ACC/AHA Guideline for the management of patients with Valvular Heart Disease: executive summary: a report of the American College of Cardiology/American Heart Association Joint Committee on Clinical Practice guidelines. J Am Coll Cardiol. 2021;77(4):450–500. 33342587 10.1016/j.jacc.2020.11.035

[6] Généreux P, Sharma RP, Cubeddu RJ, Aaron L, Abdelfattah OM, Koulogiannis KP, et al. The Mortality Burden of untreated aortic stenosis. J Am Coll Cardiol. 2023;82(22):2101–9. 37877909 10.1016/j.jacc.2023.09.796

[7] Engelman DT, Ben Ali W, Williams JB, Perrault LP, Reddy VS, Arora RC, et al. Guidelines for Perioperative Care in Cardiac surgery: enhanced recovery after surgery Society recommendations. JAMA Surg. 2019;154(8):755–66. 31054241 10.1001/jamasurg.2019.1153

[8] Noss C, Prusinkiewicz C, Nelson G, Patel PA, Augoustides JG, Gregory AJ. Enhanced recovery for cardiac surgery. J Cardiothorac Vasc Anesth. 2018;32(6):2760–70. 29503121 10.1053/j.jvca.2018.01.045

[9] Williams JB, McConnell G, Allender JE, Woltz P, Kane K, Smith PK, et al. One-year results from the first US-based enhanced recovery after cardiac surgery (ERAS Cardiac) program. J Thorac Cardiovasc Surg. 2019;157(5):1881–8. 30665758 10.1016/j.jtcvs.2018.10.164

[10] Markham T, Wegner R, Hernandez N, Lee JW, Choi W, Eltzschig HK, et al. Assessment of a multimodal analgesia protocol to allow the implementation of enhanced recovery after cardiac surgery: retrospective analysis of patient outcomes. J Clin Anesth. 2019;54:76–80. 30412813 10.1016/j.jclinane.2018.10.035

[11] Ljungqvist O. The enhanced recovery after surgery in cardiac surgery revolution. JAMA Surg. 2019;154(8):767. 31054243 10.1001/jamasurg.2019.1154

[12] Li M, Zhang J, Gan TJ, Qin G, Wang L, Zhu M, et al. Enhanced recovery after surgery pathway for patients undergoing cardiac surgery: a randomized clinical trial. Eur J Cardiothorac Surg. 2018;54(3):491–7. 29514224 10.1093/ejcts/ezy100

[13] Grant MC, Isada T, Ruzankin P, Whitman G, Lawton JS, Dodd-o J, et al. Results from an enhanced recovery program for cardiac surgery. J Thorac Cardiovasc Surg. 2020;159(4):1393–e14027. 31279510 10.1016/j.jtcvs.2019.05.035

[14] Zaouter C, Oses P, Assatourian S, Labrousse L, Rémy A, Ouattara A. Reduced length of Hospital stay for cardiac surgery—implementing an optimized perioperative pathway: prospective evaluation of an enhanced recovery after surgery program designed for mini-invasive aortic valve replacement. J Cardiothorac Vasc Anesth. 2019;33(11):3010–9. 31153719 10.1053/j.jvca.2019.05.006

[15] Grant MC, Isada T, Ruzankin P, Gottschalk A, Whitman G, Lawton JS, et al. Opioid-sparing Cardiac Anesthesia: secondary analysis of an enhanced recovery program for cardiac surgery. Anesth Analg. 2020;131(6):1852–61. 32889848 10.1213/ANE.0000000000005152

[16] Fleming IO, Garratt C, Guha R, Desai J, Chaubey S, Wang Y, et al. Aggregation of marginal gains in cardiac surgery: feasibility of a Perioperative Care Bundle for enhanced recovery in Cardiac Surgical patients. J Cardiothorac Vasc Anesth. 2016;30(3):665–70. 27321791 10.1053/j.jvca.2016.01.017

[17] Davis B, Bankhead-Kendall BK, Dumas RP. A review of COVID-19’s impact on modern medical systems from a health organization management perspective. Health Technol (Berl). 2022;12(4):815. 35371904 10.1007/s12553-022-00660-z PMC8956330

[18] French G, Hulse M, Nguyen D, Sobotka K, Webster K, Corman J, et al. Impact of hospital strain on excess deaths during the COVID-19 pandemic-United States, July 2020-july 2021. Am J Transpl. 2022;22(2):654–7. 10.1111/ajt.16645 PMC9811904 35113490

[19] Miller KA, Harkin CP, Bailey PL. Postoperative tracheal extubation. Anesth Analg. 1995;80(1):149–72. 7802273 10.1097/00000539-199501000-00025

[20] Ailawadi G, Chang HL, O’Gara PT, O’Sullivan K, Woo YJ, DeRose JJ, et al. Pneumonia after cardiac surgery: experience of the National Institutes of Health/Canadian Institutes of Health Research Cardiothoracic Surgical Trials Network. J Thorac Cardiovasc Surg. 2017;153(6):1384–e13913. 28341473 10.1016/j.jtcvs.2016.12.055 PMC5439299

[21] Sheng W, Xing QS, Hou WM, Sun L, Niu ZZ, Lin MS, et al. Independent risk factors for Ventilator-Associated Pneumonia after Cardiac surgery. J Invest Surg. 2014;27(5):256–61. 24660655 10.3109/08941939.2014.892652

[22] Hortal J, Giannella M, Pérez MJ, Barrio JM, Desco M, Bouza E, et al. Incidence and risk factors for ventilator-associated pneumonia after major heart surgery. Intensive Care Med. 2009;35(9):1518–25. 19557389 10.1007/s00134-009-1523-3

[23] Hortal J, Muñoz P, Cuerpo G, Litvan H, Rosseel PM, Bouza E, et al. Ventilator-associated pneumonia in patients undergoing major heart surgery: an incidence study in Europe. Crit Care. 2009;13(3):R80. 19463176 10.1186/cc7896 PMC2717444

[24] Crawford TC, Magruder JT, Grimm JC, Sciortino C, Conte JV, Kim BS, et al. Early Extubation: a proposed New Metric. Semin Thorac Cardiovasc Surg. 2016;28(2):290–9. 28043432 10.1053/j.semtcvs.2016.04.009

[25] Muller Moran HR, Maguire D, Maguire D, Kowalski S, Jacobsohn E, Mackenzie S, et al. Association of earlier extubation and postoperative delirium after coronary artery bypass grafting. J Thorac Cardiovasc Surg. 2020;159(1):182–e1907. 31076177 10.1016/j.jtcvs.2019.03.047

[26] Naughton C, Reilly N, Powroznyk A, Aps C, Hunt T, Hunter D, et al. Factors determining the duration of tracheal intubation in cardiac surgery: a single-centre sequential patient audit. Eur J Anaesthesiol. 2003;20(3):225–33. 12650494 10.1097/00003643-200303000-00008

[27] Cavallone LF, Vannucci A. Extubation of the difficult airway and extubation failure. Anesth Analg. 2013;116(2):368–83. 23302983 10.1213/ANE.0b013e31827ab572

[28] Helwani MA, Copeland C, Ridley CH, Kaiser HA, De Wet CJ. A 3-hour fast-track extubation protocol for early extubation after cardiac surgery. JTCVS Open. 2022;12:299. 36590715 10.1016/j.xjon.2022.07.006 PMC9801240

[29] Camp SL, Stamou SC, Stiegel RM, Reames MK, Skipper ER, Madjarov J, et al. Can timing of tracheal extubation predict improved outcomes after cardiac surgery? HSR Proc Intensive Care Cardiovasc Anesth. 2009;1(2):39. 23439795 PMC3484547

[30] Camp SL, Stamou SC, Stiegel RM, Reames MK, Skipper ER, Madjarov J, et al. Quality Improvement Program increases early tracheal extubation rate and decreases pulmonary complications and resource utilization after cardiac surgery. J Card Surg. 2009;24(4):414–23. 19583609 10.1111/j.1540-8191.2008.00783.x

[31] London MJ, Shroyer AL, Coll JR, MaWhinney S, Fullerton DA, Hammermeister KE, et al. Early extubation following cardiac surgery in a veterans population. Anesthesiology. 1998;88(6):1447–58. 9637636 10.1097/00000542-199806000-00006

[32] Flynn BC, He J, Richey M, Wirtz K, Daon E. Early Extubation without increased adverse events in high-risk Cardiac Surgical patients. Ann Thorac Surg. 2019;107(2):453–9. 30395853 10.1016/j.athoracsur.2018.09.034

[33] Wong WT, Lai VKW, Chee YE, Lee A. Fast-track cardiac care for adult cardiac surgical patients. Cochrane Database Syst Rev. 2016;2016(9). 10.1002/14651858.CD003587.pub3 PMC6457798 27616189

[34] Hirji SA, McCarthy E, Kim D, McGurk S, Ejiofor J, Ramirez-Del Val F, et al. Relationship between Hospital Surgical aortic valve replacement volume and transcatheter aortic valve replacement outcomes. JACC Cardiovasc Interv. 2020;13(3):335–43. 32029250 10.1016/j.jcin.2019.09.048 PMC8858441

[35] Hawkins AD, Strobel RJ, Mehaffey JH, Hawkins RB, Rotar EP, Young AM, et al. Operating room versus intensive care unit extubation within 6 hours after on-pump cardiac surgery: early results and hospital costs. Semin Thorac Cardiovasc Surg. 2024;36(2):195–208. 10.1053/j.semtcvs.2022.09.013 PMC10225475 36460133

[36] Shvartz V, Sokolskaya M, Petrosyan A, Ispiryan A, Donakanyan S, Bockeria L, et al. Predictors of Mortality following aortic valve replacement in aortic stenosis patients. Pathophysiology. 2022;29(1):106. 35366293 10.3390/pathophysiology29010010 PMC8955830

[37] Tjang YS, van Hees Y, Körfer R, Grobbee DE, van der Heijden GJMG. Predictors of mortality after aortic valve replacement. Eur J Cardiothorac Surg. 2007;32(3):469–74. 17658266 10.1016/j.ejcts.2007.06.012

[38] Tseng EE, Lee CA, Cameron DE, Stuart RS, Greene PS, Sussman MS, et al. Aortic valve replacement in the elderly. Risk factors and long-term results. Ann Surg. 1997;225(6):793. 9230820 10.1097/00000658-199706000-00017 PMC1190892

[39] Di Eusanio M, Fortuna D, De Palma R, Dell’Amore A, Lamarra M, Contini GA, et al. Aortic valve replacement: results and predictors of mortality from a contemporary series of 2256 patients. J Thorac Cardiovasc Surg. 2011;141(4):940–7. 20599229 10.1016/j.jtcvs.2010.05.044

[40] Moitra VK, Guerra C, Linde-Zwirble WT, Wunsch H. Relationship between ICU length of Stay and Long-Term Mortality for Elderly ICU survivors. Crit Care Med. 2016;44(4):655. 26571190 10.1097/CCM.0000000000001480 PMC4792682

[41] Nguyen Q, Coghlan K, Hong Y, Nagendran J, MacArthur R, Lam W. Factors Associated with Early Extubation after Cardiac surgery: a retrospective single-center experience. J Cardiothorac Vasc Anesth. 2021;35(7):1964–70. 33414072 10.1053/j.jvca.2020.11.051

[42] James L, Smith DE, Galloway AC, Paone D, Allison M, Shrivastava S, et al. Routine extubation in the operating room after isolated coronary artery bypass. Ann Thorac Surg. 2024;117(1):87–94. 37806334 10.1016/j.athoracsur.2023.09.031

