# Supplementary material for: Early extubation after elective surgical aortic valve replacement during the COVID-19 pandemic
Source: J Cardiothorac Surg. 2024 Aug 24;19:490. doi: 10.1186/s13019-024-02989-0 (PMC11344404; doi:10.1186/s13019-024-02989-0)
Supplement: Supplementary file 1 — Supplementary Material 1 [file 13019_2024_2989_MOESM1_ESM.docx]

**Supplements**

Supplement Table 1. LOS in ICU and in the hospital of patients in the ITN group.

|  | **Postoperative pneumonia (ITN) (n=41 )** | **No postoperative pneumonia (ITN) (n=9)** | **P Value** |
| --- | --- | --- | --- |
|  |  |  |  |
| ICU LOS (days) | 4 (2-6) | 25 (11-63) | < 0.001* |
| Hospital LOS (days) | 10 (8-15) | 34 (13-39) | < 0.001* |

Patients in the ITN group experience longer LOS in both, the ICU and the hospital. LOS = length of stay; ICU = intensive care unit.
